# T24. REDOX REGULATORS AND OXIDATIVE STRESS IN SCHIZOPHRENIA

**DOI:** 10.1093/schbul/sby016.300

**Published:** 2018-04-01

**Authors:** Havard Bentsen, Dag Solberg, Helge Refsum, Ole Andreassen

**Affiliations:** 1 Haukeland University Hospital; 2 Diakonhjemmet Hospital; 3 Oslo University Hospital

## Abstract

**Background:**

Elevated levels of oxidative stress have been reported in schizophrenia and may play a role in the underlying psychopathology. The antioxidant defense system may be disrupted in schizophrenia. We have earlier shown how levels of membrane polyunsaturated fatty acids (PUFA) change during the course of schizophrenia, increasing from a low level in a subgroup, remaining stable in another. Here, we aimed at comparing levels of redox regulators, oxidative stress, and related genotypes in schizophrenia patients and healthy controls, and at identifying how these biomarkers are related to membrane fatty acids and clinical characteristics in acute and chronic phase of schizophrenia.

**Methods:**

Patients with schizophrenia spectrum disorders (n=55) examined during an acute phase and five years later during a chronic phase, and healthy controls (n=51) were included. We assessed blood levels of redox regulators [(alpha-tocopherol, bilirubin, uric acid, glutathione (GSH), glutathione peroxidase (GPx)], glutathione reductase (GR), markers of oxidative stress [F2-isoprostane, reactive oxygen metabolites (D-ROMs)] and PUFA. We examined genotypes and gene expression related to glutamate cysteine ligase (GCL), the rate-limiting enzyme of GSH synthesis, and its catalytic (GCLC) and modulator (GCLM) subunits. Links between redox measures and Positive and Negative Syndrome Scale (PANSS) were studied.

**Results:**

In the chronic phase, levels of alpha-tocopherol (p=0.03) and bilirubin (p<0.001) were lower, while uric acid (p=0.02) was higher in patients than in controls. In patients, the levels of alpha-tocopherol were higher in the acute phase than in the chronic phase (p=0.001). However, the changes depended on the PUFA levels in the acute phase. In the low PUFA group, alpha-tocopherol levels remained stable, whereas in the high PUFA group, they decreased to those of the low PUFA group. Levels of D-ROMs were linked to PUFA (r=0.39, p=0.007) and long-chain PUFA (r=0.42, p=0.003) in controls but not in patients.

There was no significant difference in the distribution of GCLC genotypes between patients and controls. Compared to other GAG trinucleotide-repeat (TNR) genotypes, 7/9 GAG genotype was linked to higher gene expression of GCL (mRNA GCLC, p=0.049; mRNA GCLM, p=0.02), higher levels of GSH in blood (p=0.02) and higher GR activity in blood (p=0.007). Only when combined with C-129T high risk genotype, the 7/7 GAG genotype induced a strong reduction of CGLC expression (p=0.045, bootstrapping 10000 samples). Gene expression related to glutathione synthesis was non-significantly (p=0.10–0.19) higher among patients. Levels of long-chain PUFA were significantly positively linked to gene expression of glutathione related enzymes.

**Discussion:**

The findings of abnormal levels of alpha-tocopherol, bilirubin, uric acid and glutathione synthesis in the chronic phase of schizophrenia indicate oxidative stress. Dysregulation of antioxidant defenses may be involved in the pathophysiology of schizophrenia and warrants further experimental studies.

